# MRI CNS Atrophy Pattern and the Etiologies of Progressive Ataxias

**DOI:** 10.3390/tomography8010035

**Published:** 2022-02-08

**Authors:** Mario Mascalchi

**Affiliations:** Department of Clinical and Experimental Biomedical Sciences “Mario Serio”, University of Florence, Viale GB Morgagni 50, 50134 Florence, Italy; mario.mascalchi@unifi.it

**Keywords:** ataxia, MRI, CNS

## Abstract

MRI shows the three archetypal patterns of CNS volume loss underlying progressive ataxias in vivo, namely spinal atrophy (SA), cortical cerebellar atrophy (CCA) and olivopontocerebellar atrophy (OPCA). The MRI-based CNS atrophy pattern was reviewed in 128 progressive ataxias. A CNS atrophy pattern was identified in 91 conditions: SA in Friedreich’s ataxia, CCA in 5 acquired and 72 (24 dominant, 47 recessive,1 X-linked) inherited ataxias, OPCA in Multi-System Atrophy and 12 (9 dominant, 2 recessive,1 X-linked) inherited ataxias. The MRI-based CNS atrophy pattern may be useful for genetic assessment, identification of shared cellular targets, repurposing therapies or the enlargement of drug indications in progressive ataxias.

## 1. Introduction

Progressive ataxias are a group of many uncommon yet often very disabling diseases, which can be inherited or acquired. The average prevalence of recessive hereditary ataxias is 3.3/10^5^ and of dominant hereditary ataxias is 2.7/10^5^ [1]. Although the world frequency of each type of progressive ataxia is ethnically and regionally inhomogeneous, reflecting the clustering effect of inherited conditions, Friedreich ataxia (FRDA) and spinocerebellar ataxia type 3 (SCA3) are the most common types of recessive and dominant ataxia, respectively [1]. Multi-System Atrophy cerebellar type is the most common type of acquired ataxia [2].

Neuropathological studies between 1877 and 1922 [3,4,5] recognized three archetypes of progressive ataxias based on the predominant distribution of severity of the neuronal systems damage among the spinal cord, brainstem and cerebellum, namely spinal atrophy (SA), cortical cerebellar atrophy (CCA) and olivopontocerebellar atrophy (OPCA).

Since the last decade of the twentieth century, genetic and molecular genetic studies revealed that an increasing number of mutations of different protein-coding genes can underlie dominant, recessive and X-linked progressive ataxias, and this allows us to classify inherited ataxias according to presumed molecular pathogenesis [6,7,8]. Moreover, screening has revealed that genetic causes are also involved in up to 22% of patients presenting with sporadic progressive ataxia [2,9,10,11,12,13,14]. This justifies the systematic search of possible gene mutations in such patients. As a matter of fact, the number of “idiopathic” cases of progressive ataxia is decreasing, but a high number of potential candidate genes must ultimately be assessed in the diagnostic work of the single patient [8,15,16,17,18,19].

In particular, the introduction of modern instruments for gene sequencing (next generation sequencing (NGS)) has made it easier to reach a genetic diagnosis in cases with typical phenotypes using targeted multigene panels or whole-exome sequencing. Moreover, whole-genome sequencing has further expanded the genetic causes of ataxia in patients with atypical phenotypes [15,18,20,21]. Not unexpectedly, once sporadic causes have been excluded, it is tempting to use NGS techniques as the first diagnostic step in patients with progressive ataxia, according to a “reverse phenotyping” approach in which phenotype characterization follows genetic results [15,20]. However, the large number of repeat-expansion disorders underlying progressive ataxias is not well covered by NGS techniques [8,22,23], and it has been emphasized that proper classification and phenotype characterization according to a “phenotyping first” approach is still fundamental to offer the patient custom gene testing [8,24].

Magnetic resonance imaging (MRI) is a safe technique and constitutes a fundamental tool for both differential diagnosis of the causes of acute and subacute ataxia [25] and characterization of patients with progressive chronic ataxia with in vivo demonstration of three archetypal CNS atrophy patterns demonstrated by pathology [26,27,28,29]. The contribution of conventional MRI to the diagnosis in patients presenting with the most frequent acquired or inherited progressive ataxias has recently been re-assessed [30,31,32,33], and a variable combination of distributed atrophy pattern and signal changes in the brain and spinal cord has been recognized as valuable support for diagnosis and inserted in the diagnostic workflow and algorithms [24,30,32]. However, progressive ataxias often show non-specific and sometimes overlapping MRI findings which seldom allow per se a definite diagnosis.

Since the pattern of CNS atrophy on MRI is associated with a definite list of diagnostic possibilities in inherited and acquired progressive ataxias, it is possible to group these diseases according to the three archetypes [28,29]. This clumping approach might primarily help to define the patient’s phenotype and contain the need for costly genetic panels by narrowing the etiological hypotheses to those belonging to each category [34]. The purpose of this review is to update the relationship between the MRI CNS atrophy pattern and etiologies of progressive ataxias that was originally proposed for 28 conditions [28] and here is extended to 128 progressive ataxias.

## 2. Materials and Methods

### 2.1. Classification and Nomenclature of Progressive Ataxias

The classification of ataxias is complex and, in the case of inherited ataxias, following NGS introduction, subject to continuous additions. To establish an updated relationship between the MRI CNS atrophy pattern and etiology in progressive ataxias, the following mixed procedure to select the diseases to be evaluated was set to be inclusive while defining some borders.

Attention was drawn on progressive subacute and chronic ataxias, namely those with onset in months or years, whereas acute and episodic ataxias were excluded [35]. Four main types of progressive ataxias were identified: acquired, dominantly inherited, recessively inherited, and X-linked. The constant updates of the list of inherited ataxias and the variable names attributed to each entity suggest that the classification of inherited ataxias should adopt the Online Mendelian Inheritance in Man (OMIM) nomenclature and number. However, a survey at OMIM in October 2021 using “ataxia” as a “keyword” yielded 1440 entities, whereas 67 entities were obtained using “SCA” (spinocerebellar ataxia dominant), 307 using “SCAR” (spinocerebellar ataxia recessive), and 5 using “X-linked ataxias” (https://www.omim.org accessed on 26 October 2021). The huge number of recessive ataxias labelled as “SCAR” in the OMIM system overwhelms any reasonable and useful analysis. As a matter of fact, in recent years, two proposals for the classification of recessive ataxias based on the frequency and predominance of the cerebellar ataxia symptoms and signs in recessive diseases were proposed. They collected 58 [8] and 92 [6] entities, respectively.

It was decided to assume the 58 recessive ataxias recognized as primary autosomal recessive cerebellar ataxias by the Ataxia Task Force report in 2019 as a reference base for our purpose [8]. This was preferred to the list of 92 entities listed by the International Parkinson and Movement Disorder Society Task Force on Classification and Nomenclature of Genetic Movement Disorders [6] because the latter excluded priori entities in which purely sensory ataxia is prominent and cerebellar symptoms and signs are lacking. Moreover, the many multisystem recessive disorders which can present ataxia as a clinical feature [8] or entities in which ataxia is combined with other often prominent movement disorders [6] were beyond the scope of the present review and were excluded. However, differently from the Ataxia Task Force [8], entities labeled as SCAR or X-linked ataxia in OMIM that, although described in a single family, are well characterized for the causative genetic abnormality (as recognized in OMIM with the # suffix) were included, whereas entities with undefined genetic abnormalities (indicated in OMIM with the % suffix), such as SCA4, SCA9, SCA18, SCA25, SCA30, SCA32, SCAR3, SCAR6 and SCAX5, were excluded. In addition, purely malformative conditions, such as the Joubert syndrome or Dandy–Walker malformation were excluded, and only the mitochondrial diseases contained in the Ataxia Task Force 2019 list or in OMIM under the “SCAR” label were considered.

Finally, the list of inherited ataxias was integrated with the cerebellar Ataxia, neuropathy and vestibular areflexia syndrome (CANVAS) (OMIM 614575), the fragile X tremor ataxia syndrome (FXTAS) (OMIM 300623) and the PRPS1 gene mutation [21] found in a PubMed survey from October 2021.

Overall, the list of progressive ataxias covered in this review is not exhaustive but representative of most of the less rare and better genetically characterized entities, yielding a total of 128 diseases: 11 acquired and 117 inherited (42 dominant, 72 recessive and 3 X-linked).

### 2.2. Definition of the Archetypes on MRI

**Figure 1:**
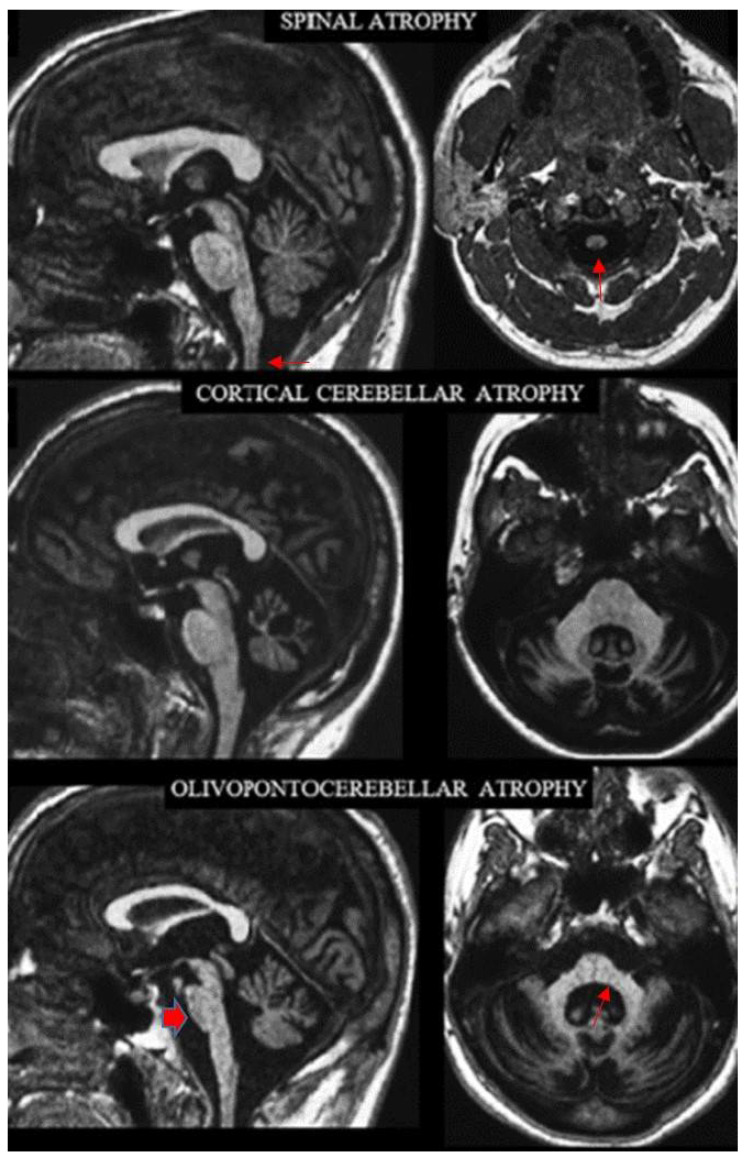
Sagittal (left column) and axial (right column) T_1_-weighted MR images in three exemplificative patients show the three CNS atrophy patterns underlying progressive ataxias (modified by ref. [29]). The typical features of spinal atrophy (SA), namely thinned medulla and cervical spinal cord (arrows) with normal volume of the pons and cerebellar vermis, are observed in a patient with Friedreich ataxia (top). Loss of bulk of the vermis and cerebellar hemispheres with enlarged interfolia spaces but normal volume of the pons, middle cerebellar peduncles and of the cervical spinal cord are the hallmark of cortical cerebellar atrophy (CCA) in a patient with sporadic adult-onset ataxia (SAOA) (mid). Atrophy of the brainstem, more pronounced in the inferior portion of the basis pontis (arrowhead), of the vermis, of the middle cerebellar peduncles (arrow) and of the cerebellar hemispheres characterizes olivopontocerebellar atrophy (OPCA) in a patient with SCA2 (bottom). Note in the bottom images the thinning of the superior cervical medulla that can be observed in SCA2 as result of secondary axonal and trans-synaptic degeneration.

The names of the three archetypes of atrophy convey the distribution of the predominant loss of bulk in the CNS underlying progressive ataxias. It affects the spinal cord in SA, the cortical cerebellum in CCA and the brainstem and cerebellum in OPCA. Visual assessment of conventional MRI allows recognition of these patterns (Figure 1). The three atrophy patterns can also be more objectively defined according to the distribution of abnormally decreased bidimensional (linear or area) measurements in sagittal and axial MR images [26], which were not used in this review. Patients with SA have abnormal values for the cervical spinal cord but no additional abnormal values, except for the fourth ventricle and the medulla. Patients with CCA have abnormal values for the cerebellar vermis or hemispheres but no additional abnormal values except for the fourth ventricle and the middle cerebellar peduncle. Patients with OPCA have abnormal values for the cerebellar vermis or hemispheres and at least two abnormal values within the basis pontis, middle cerebellar peduncles and medulla oblongata.

In reviewing the MR images of each disease, the distinction indicated by Poretti and Boltshauser [36] between cerebellar “hypoplasia” and “atrophy” was applied. Regrettably, the two terms are sometimes used interchangeably, leading to confusion and possible misdiagnosis. Cerebellar hypoplasia refers to cerebellum with a reduced volume which is stable over time. Cerebellar atrophy is defined as a cerebellum in a posterior fossa with normal size, which displays enlarged fissures and interfolial spaces secondary to irreversible loss of tissue due to progressive disease or a single injury. Admittedly, the distinction between cerebellar hypoplasia and cerebellar atrophy based on a single examination can be difficult. However, to differentiate the OPCA pattern from “pontocerebellar hypoplasia”, we also considered, on the one hand, the typically flattened shape of the basis pontis in sagittal images and the “pointed” shape in the coronal images of the middle cerebellar peduncles, which are observed in OPCA [37]. On the other hand, the “dragonfly” or “butterfly” appearance of the cerebellum on coronal MR images that is peculiar of pontocerebellar hypoplasia [38], with the flattened cerebellar hemispheres representing “the wings”, is associated with a less pronounced (dragonfly) or proportional (butterfly) vermis size decrease.

Since computed tomography (CT) does not allow assessment of the brainstem and spinal cord, it was not considered to attribute a CNS atrophy pattern underlying progressive ataxias.

### 2.3. Review of the MRI Features

This reviewer has 35-years of experience in neuro-MRI with a specific interest in ataxias [28,29,39,40].

To update the relationship between the CNS atrophy pattern and etiologies of acquired progressive ataxias he consulted three review articles [30,31,32] and the source papers cited in the reviews themselves [41]. For the inherited progressive ataxias, he reviewed all the MRI images or the descriptions of the MRI features contained in all papers referenced in the Ataxia Task Force article on recessive ataxias [8] and in the papers on inherited ataxias accessible through OMIM using the links provided under each disease category. Additionally, using access through PubMed, he scrutinized further MRI images or descriptions in recent papers dealing with progressive ataxias [42,43,44,45,46], reporting cases with new mutations not yet included in OMIM [21]. Reviewed images were predominantly sagittal and axial T_1_ or T_2_-weighted images, which display changes in size and signal of the cerebellum, brainstem and spinal cord well.

The review yielded seven possible outcomes: (1) Typical CNS atrophy pattern among SA, CCA and OPCA; (2) Ponto-cerebellar hypoplasia; (3) Generalized CNS atrophy; (4) Absence of CNS atrophy; (5) Impossible evaluation (when neither MR images nor description of the MRI features were available); (6) Uncertain CNS atrophy pattern; (7) Progressive ataxias in which characteristic MRI signal abnormalities are prominent as compared to mild or absent atrophy and presumably explained the progressive ataxia.

Based on the substantial heterogeneity and disperse distribution of the source data, no statistical analysis was performed.

## 3. Results

A disease-associated MRI CNS atrophy pattern was identified in the majority, 71% (91/128), of the diseases.

Table 1 show the distribution of MRI based atrophy patterns among the acquired, inherited and progressive ataxias.

The SA pattern was identified only in Friedreich’s ataxia. The CCA pattern was observed in 77 conditions: 5 acquired (alcoholic cerebellar degeneration, gluten ataxia, anti-glutamic acid decarboxylase ataxia, paraneoplastic cerebellar degeneration and sporadic adult-onset ataxia/idiopathic late-onset cerebellar ataxia) and 72 (24 dominant, 47 recessive, 1 X-linked) inherited ataxias. The OPCA pattern was recognized in the multi-system atrophy cerebellar type, and 12 (nine dominant, two recessive, one X-linked) inherited ataxias.

MRI showed pontocerebellar hypoplasia in three recessively inherited progressive ataxias, namely cerebellar ataxia mental retardation with/out quadrupedal locomotion type 1 (CAMRQ1, OMIM 224050), type 2 (CAMRQ2, OMIM 610185) and type 3 (CAMRQ3, OMIM 613227).

A generalized CNS atrophy was observed in two dominantly inherited diseases, i.e., SCA17 (OMIM 607136) and autosomal dominant cerebellar ataxia, deafness and narcolepsy (OMIM 604121).

No evidence of CNS atrophy was reported in four inherited ataxias: three dominant including SCA41 (OMIM 616410), SCA46 (OMIM 617770) and spastic ataxia type 1 (SPAX1, OMIM 108600), and one recessive, namely SCAR23 (OMIM 616939).

The presence and type of CNS atrophy was impossible to define in two recessive conditions, namely ataxia telangiectasia-like disease (OMIM 604391) and spastic ataxia type 4 (SPAX4, OMIM 613672).

The CNS atrophy pattern was uncertain for 11 progressive ataxias. This reflected the small number of patients examined with MRI in two dominant ataxias, i.e., SCA40 (OMIM 616053) and SCA45 (OMIM 617769), and two recessive ataxias, i.e., SCAR12 (OMIM 614322) and SCAR15 (OMIM 615705). In addition, uncertainty surrounded the pattern inthree recessive ataxias in which the MRI findings were heterogeneous or non-specific, including for instance cerebral periventricular hyperintensities in T_2_-weighted images. These included SeSAME syndrome (OMIM 612780), spastic ataxia type 2 (OMIM 611302) and spastic paraplegia 5A (OMIM 270800). In four further instances, the CNS atrophy pattern was uncertain because different patterns among SA, CCA and OPCA were reported comprising early-onset cerebellar ataxia (EOCA) with retained tendon reflexes, ataxia with vitamin E deficiency (AVED) (OMIM 277460), SCAR27 (OMIM 618369/618128) and cerebellar ataxia, neuropathy and vestibular areflexia syndrome (CANVAS) (OMIM 614575).

Finally, fifteen progressive ataxias were characterized by MRI signal abnormalities in T_2_-weighted images that were predominant over the absent or mild atrophy of the CNS structures. They are detailed in Table 2. The acquired causes besides tumors, which show variable signal changes, include Creutzfeldt–Jakob disease in which basal ganglia and cortex show hyperintensity in T_2_-weighted images and decreased diffusion, siderosis in which CNS surfaces show low signal rim in T_2_ or T_2*_-weighted images, and Vit B12 deficiency in which the posterior columns of the spinal cord appear hyperintense in T_2_-weighted images.

Inherited causes included two dominant ataxias, namely ataxia-pancytopenia syndrome (OMIM 159550), in which the cerebral white matter (WM) is hyperintense in T_2_-weighted images, and SCA20 (OMIM 608687), in which the cerebellar dentate nuclei show low signal due to calcifications in T2 or T2*-weighted images.

Hyperintensities in T_2_-weighted images are observed in seven recessive ataxias. They involve the cerebral WM in five recessive ataxias including cerebrotendinous xanthomatosis (CTX) (OMIM 213700), 2-hydroxic glutaric aciduria (OMIM 236792), hypomyelinating leukodystrophy type 2 (HLD2) (OMIM 608804), leukoencephalopathy with brainstem and spinal cord involvement and lactate elevation (LBSL)(OMIM 611105), and SCAR4/SCA24 (OMIM 607317). Additional hyperintense areas in T_2_-weighted images are observed in the peridentate cerebellar WM in CTX and HLD2, in the cerebral and cerebellar peduncles, brainstem and spinal cord in LBSL, and in the basal ganglia in SCAR4/SCA24. In leukoencephalopathy with ataxia (OMIM 615651), the hyperintensity in T_2_-weighted images involves the internal capsule, cerebral peduncles and middle cerebellar peduncles and is associated with restricted diffusion, whereas in the sensory ataxic neuropathy, dysarthria and ophtalmoparesis (SANDO) (OMIM 607459) it involves the thalami, middle cerebellar peduncles and the cerebellar WM.

A lack of normal myelination is characteristic of hypomyelinating leukodystrophy type 4 (OMIM 612233).

In X-linked fragile-X tremor ataxia syndrome (FXTAS) (OMIM 300623), hyperintensity in T_2_-weighted images is typically observed in the cerebral WM, splenium of the corpus callosum and middle cerebellar peduncles.

## 4. Discussion

A review of the images and descriptions in the published cases and series allowed us to recognize a typical MRI-based CNS atrophy pattern in the majority (71%; 91/128) of progressive ataxias.

### 4.1. Definite CNS Atrophy—Etiological Relationship

The SA pattern was exclusively observed in FRDA. The CCA pattern was observed in several acquired ataxias (alcoholic cerebellar degeneration, gluten ataxia, anti-glutamic acid decarboxylase ataxia, paraneoplastic cerebellar degeneration and sporadic adult-onset ataxia/idiopathic late-onset cerebellar ataxia), in many dominant ataxias (SCA5, SCA6, SCA8, SCA10, SCA11, SCA12, SCA13, SCA14, SCA15/16, SCA19/22, SCA20, SCA21, SCA26, SCA27, SCA28, SCA31, SCA35, SCA37, SCA38, SCA42, SCA43, SCA44, SCA47 and SCA48), all recessive ataxias with the exception of Boucher–Neuhauser Syndrome (BNS) and SCAR7 and in one X-linked ataxia (SCAX1).

The OPCA pattern was identified as MSA-C in a few dominant ataxias (SCA1-3, SCA7, SCA34, SCA36, DRPLA), two recessive ataxias (BNS and SCAR7) and the X-linked ataxia associated with the PRPS1 gene mutation.

### 4.2. Pontocerebellar Hypoplasia

MRI showed pontocerebellar hypoplasia in all three recessive diseases characterized by progressive ataxias and mental retardation with/out quadrupedal locomotion type 1–3 (CAMRQ1, CAMRQ2 and CAMRQ3). On the other hand, unfortunately, in some instances, the term “hypoplasia” was used to describe MRI findings of probable CAA as in SCAR25, SCAR28, SCAR31 and SCAX1.

### 4.3. Uncertain CNS Atrophy—Etiological Relationship

The CNS atrophy pattern was uncertain in 11 progressive ataxias. A small number of patients were examined with MRI in SCA40, SCA45, SCAR12 and SCAR15, and heterogeneous and non-specific MRI findings were observed or reported in SeSAME syndrome, spastic ataxia type 2 and spastic paraplegia 5A.

More articulated are the reasons for the inclusion of four further progressive ataxias in the category of uncertain CNS atrophy patterns, namely EOCA, AVED, SCAR27 and CANVAS. EOCA is recognized as a heterogeneous sporadic condition that was initially considered separated from FRDA. However, FRDA patients can present with an EOCA phenotype [60]. Initially, a CCA pattern was described in patients with EOCA, possibly contributing to the differentiation with FRDA [61]. However, subsequent reports described atrophy of the brainstem and spinal cord [27,62]. AVED is a recessive condition for which relatively few data on CNS morphology are available. No brain MRI abnormality was observed in some patients, whereas cerebellar atrophy was reported in others [63]. Moreover, in the only AVED patient examined with spinal MRI, no abnormality was found [64], but a decreased size of the upper cervical cord was apparent in a case shown by Heidelberg et al. [30]. Hence, despite the reasonable assumption of a SA pattern in AVED reflecting the sensory ataxia and the clinical similarity with FRDA [8,29], no definitive evidence of it has been provided so far. In the original description of SCAR27, a CCA pattern was recognized in two unrelated patients [65]. However, in a further patient, it was accompanied by atrophy of the pons and midbrain [42]. CANVAS is a recessive condition with marked clinical heterogeneity that has recently been recognized as responsible for apparently sporadic progressive ataxia [14,45,46,66]. CANVAS showed a pronounced heterogeneity concerning the distribution of the CNS atrophy in inherited progressive ataxia. In fact, the CCA pattern was observed in the majority of patients with CANVAS [43,44], but it was combined with atrophy in the spinal cord in some patients who were examined with cervical spine MRI [44], and also an OPCA pattern was reported in a few patients [45,46].

### 4.4. Progressive Ataxias Characterized by MRI Signal Changes

Some progressive ataxias are characterized by signal changes better demonstrated by T_2_-weighted images, which, irrespective of the CNS atrophy severity and distribution patterns, can considerably help in identifying them. These include acquired and inherited causes. The signal changes in the acquired forms have been reviewed elsewhere [31,47,48]. The inherited forms include two dominant ataxias, namely ataxia-pancytopenia syndrome (OMIM 159550 [49], and SCA20 (OMIM 608687) [50], seven recessive ataxias, namely cerebrotendinous xanthomatosis (CTX) (OMIM 213700) [51], 2-hydroxic glutaric aciduria (OMIM 236792) [52], hypomyelinating leukodystrophy type 2 (HLD2) (OMIM 608804) [53], hypomyelinating leukodystrophy type 4 (OMIM 612233) [54], leukoencephalopathy with brainstem and spinal cord involvement and lactate elevation (LBSL) (OMIM 611105)[55], leukoencephalopathy with ataxia (OMIM 615651) [56], sensory ataxic neuropathy, dysarthria and ophtalmoparesis (SANDO) (OMIM 607459) [57], SCAR4/SCA24 (OMIM 607317)[58] and the X-linked fragile-X tremor ataxia syndrome (FXTAS) (OMIM 300623) [59].

The signal in T_2_-weighted images is symmetrically increased in the majority of these inherited ataxias, variably involving the cerebral WM, basal ganglia and thalami, cerebral peduncles, cerebellar peduncles, cerebellar WM, brainstem and spinal cord. The distribution is characteristic in HLD2 (OMIM 608804) [53], (LBSL) (OMIM 611105) [55], leukoencephalopathy with ataxia (OMIM 615651) [56], (SANDO) (OMIM 607459) [57], SCAR4/SCA24 (OMIM 607317)[58] and X-linked fragile-X tremor ataxia syndrome (FXTAS) (OMIM 300623) [59]. More distinctive is the symmetrically decreased signal in T2 or T2*-weighted images reflecting iron deposition in the surface of the brain in siderosis [31] and calcification of the dentate nuclei in SCA20 (OMIM 608687) [50].

### 4.5. Clinical, Diagnostic and Other Implications

As expected, the distribution of the loss of bulk matching one of the CNS atrophy patterns is in line with the constellation of clinical symptoms and signs in patients with progressive ataxias [6,7]. For instance, most of the so-called pure cerebellar dominant ataxias (ADCA type III) show a CCA pattern, whereas dominant ataxias with additional extra-cerebellar symptoms and signs (ADCA type I) show an OPCA pattern. FRDA, in which there is prominent sensory involvement, shows a SA pattern and spinal cord atrophy.

Overall, this review substantially confirms the relationship between the MRI CNS atrophy pattern and etiologies in progressive ataxias [28]. Changes with respect to the 2008 classification include the displacement of SCA13 from the OPCA to CCA pattern following the report by Subramony et al. [67] in a large family and three index cases, and displacement of SCA17 from CCA to a generalized CNS atrophy pattern.

Obviously, for diagnostic purposes, the relationship between the MRI CNS atrophy pattern and etiologies in progressive ataxias must be integrated with other clinical and laboratory data and, in the case of inherited progressive ataxias, with ethnicity and geographical distribution [8,68]. However, before this use for the diagnosis in a single patient, two notes of caution are worthy.

First, it is conceivable that in advanced stages of different ataxias, generalized atrophy of the CNS, including cerebellum, brainstem, spinal cord and cerebrum, takes place as a result of secondary axonal and trans-synaptic degeneration with the waning of differences between SA, CCA and OPCA patterns. This possibility is confirmed by the occurrence of cerebellar cortex atrophy in advanced cases of FRDA, the prototype of SA [69], atrophy of the spinal cord in SCA1 and SCA3 that are typical examples of OPCA [70,71], and of pontine atrophy in SCA13 and SCA36, two conditions characterized by CCA in the early phases [67,72,73]. In addition, the correlation in CANVAS between disease duration and MRI evidence of brainstem atrophy is in line with this hypothesis [46]. However, the proposed relationship is generally valid for the early and full clinical manifestation of diseases, and data in pre-symptomatic patients with dominantly transmitted ataxia show that early loss of bulk involves the cerebellum and pons in SCA1 and SCA2, two examples of OPCA pattern [74], but the cerebellum alone in SCA48 [75] that is an example of CCA.

Second, theoretically, in line with the known phenotype and genotype heterogeneity of inherited ataxias [8], it cannot be excluded that different CNS atrophy patterns can correspond to the same disease entity.

Beyond the diagnostic purpose, two additional potential consequences of awareness of the relationship between the three MRI-based CNS atrophy patterns and the etiologies of progressive ataxias can be envisioned. First, it may contribute to identifying shared cellular targets or metabolic pathways for diseases exhibiting the same archetypal CNS atrophy pattern, thus improving our understanding of physiopathological mechanisms of progressive ataxias [8]. Second, from a therapeutic perspective, it may facilitate the repurposing of drugs or enlarge indications for inherited or acquired ataxia diseases sharing the same MRI atrophy distribution pattern (and similar distribution of neuronal systems damage) and corresponding patients [8], as was attempted in cases of CCA [76].

### 4.6. Limitations

The updated classification of progressive ataxias according to the MRI-based CNS atrophy pattern is not definitive, and further amplifications and modulations are required. Moreover, this review certainly has some limitations. First, some progressive disorders which may present with ataxia as a relevant clinical feature were arbitrarily excluded. For them, reference is made to other reviews [6,8,31]. Second, the visual assessment of the published MR images is subjective and was performed by a single observer. This can be overcome by an expert panel blind reviewers. However, discrepancies between the MRI CNS atrophy pattern reported by the authors and the one attributed by re-evaluating the published MR images was essentially restricted to the few instances concerning the difficult distinction between “hypoplasia” and “atrophy” [36]. Third, symmetric signal hyperintensities in T_2_ or T_2*_-weighted MR images reflecting Wallerian degeneration of WM tracts can accompany the SA and OPCA pattern and contribute to their full MRI pictures [37,39]. However, these signal changes are usually observed in advanced phases of the diseases when loss of bulk is already appreciable and are more sensitive to the operator variability and the technical details of the sequence and magnetic field strength. For these reasons, they were not considered to define the SA or OPCA patterns. In addition, the hyperintensity in T_2_-weighted images of the dentate signal that was reported in some inherited ataxias as SCA48 [77] and SPG7 [32] was not accounted for. Fourth, MRI quantitative techniques to evaluate the microstructure of the WM, as diffusion-weighted or diffusion tensor imaging [78,79,80], or of the dentate and other gray matter nuclei, as T_2_ -relaxometry [81] or susceptibility-weighted imaging [82] were not taken into account.

## 5. Conclusions

In line with the neuropathological discoveries of the XIX and XX centuries, MRI confirms today that there are three fundamental distribution patterns of CNS atrophy underlying progressive ataxias in vivo. They are SA, CCA and OPCA and can be inherited or acquired. Although the present trend driven by molecular genetics advances is to split progressive ataxias into hundreds of sometimes very rare conditions, a simple clumping of them according to the MRI-based CNS atrophy pattern is possible and might help diagnosis, possibly improve physiopathology understanding and may even cause future studies to rethink therapies for these uncommon but disabling diseases.

## Figures and Tables

**Table 1:** MRI CNS atrophy pattern and etiologies in progressive ataxias.

MRI CNS Atrophy Pattern		Etiology	
Spinal Atrophy (SA)	Cerebellar Cortical Atrophy (CCA)	Olivopontocerebellar Atrophy (OPCA)	
	Alcoholic cerebellar degeneration Gluten ataxia Anti-GAD ataxia Paraneoplastic cerebellar degeneration SAOA/ILOCA	MSA-C	Acquired
-	SCA5(600224), SCA6(183086) SCA8(608768), SCA10(603516) SCA11(604432), SCA12(604326) SCA13(605259), SCA14(605361) SCA15/16(606658) SCA19/22(607346), SCA20(608687) SCA21(607454), SCA26(609306), SCA27(609307) SCA28(610246), SCA31(117210) SCA35(613908), SCA37(615945) SCA38(615957), SCA42(616795) SCA43(617018) SCA44(617691), SCA47(617931) SCA48(618093)	DRPLA(125370) SCA1(164400), SCA2(183090) SCA3(109150), SCA7(164500) SCA23(610245), SCA29(117360), SCA34(133190), SCA36(614153)	Inherited Dominant
FRDA (229300)	AOA1 (208920) AOA2/SCAN2/SCAR1 (606002) AOA4(616267) ARCA1/SCAR8 (610743) ARCA2(612016) ARSACS (270550) AT (208900) Cayman ataxia(601238) CLN11(614706) GHS(212840) Gillespie syndrome(206700) HLD7(607694), HLD8(614381) Mitochondrial complex IV deficiency(220110) MTDP7 syndrome(271245) MGCA5(610198) MMS(248800) PBD6B(614871) PHARC(612674) Progressive myoclonic epilepsy 6(614018) SCAN1(607250), SCAN3(618387) SCAR2(213200) SCAR5(25130) SCAR9(612016), SCAR10(613728) SCAR11(614229) SCAR13(614831), SCAR14(615386) SCAR16(615768), SCAR17(616127) SCAR18(616204), SCAR19(616291) SCAR20(616354), SCAR21(616719) SCAR22(616948), SCAR24(617133) SCAR25(617584), SCAR26(617633) SCAR28 (618800), SCAR29(619389) SCAR30(619405), SCAR31(619422) SPAX5(614487) SPG7 (607259), SPG46(614409) SPG76(616907), SPG79(615491)	BNS (215470/2754) SCAR7(609270)	Inherited Recessive
-	SCAX1(302500)	PRPS1 Gene Mutation	Inherited X-linked

Number in parenthesis corresponds to OMIM code. **Abbreviations**: Anti-GAD ataxia = anti-glutamic acid decarboxylase; AOA = ataxia with oculomotor apraxia; ARCA = autosomal recessive cerebellar ataxia; ARSACS = autosomal recessive spastic ataxia of Charlevoix–Saguenay; AT = ataxia telangiectasia; BNS = Boucher–Neuhauser syndrome; CLN = ceroid lipofuscinosis neuronal; DRPLA = dentatorubral-pallidoluysian atrophy; EOCA = early-onset cerebellar ataxia with retained tendon reflexes; FRDA = Friedreich’s ataxia; GAD = glutamic acid decarboxylase; ILOCA = idiopathic late-onset cerebellar ataxia; GHS = Gordon Holmes syndrome; HLD = hypomyelinating leukodystrophy; MGCA5 = 3 methyglutaconic aciduria 5; MMS = Marinesco–Sjogren syndrome; MSA-C = multi-system atrophy cerebellar type; MTDP7 = mitochondrial DNA depletion syndrome 7; PHARC = polyneuropathy hearing loss ataxia retinitis pigmentosa and cataract; PBD = peroxisome biogenesis disorder; PRPS1 = phosphoribosyl pyrophosphate synthetase 1; SAOA = sporadic adult onset ataxia; SCA = spino-cerebellar ataxia; SCAN = spino-cerebellar ataxia with axonal neuropathy; SCAR = spino-cerebellar ataxia recessive; SPG = spastic paraplegia; SPAX = spastic ataxia; XSCA = X-linked recessive spino-cerebellar ataxia.

**Table 2:** Progressive ataxias characterized by predominant MRI signal changes.

Etiology		Signal Changes	References Number
Acquired	Tumors	Variable	[47]
	Kreutzfeld–Jakob disease	Basal ganglia and cortex hyperintensity in T_2_-weighted images and decreased diffusion	[48]
	Siderosis	Low signal rim of CNS surfaces in T_2_ or T_2*_-weighted images	[31]
	Vit B12 deficiency	Hyperintense posterior columns of the spinal cord in T_2_-weighted images	[31]
Dominantly inherited	ATXPC(159550)	Hyperintense cerebral WM in T_2_-weighted images	[49]
	SCA20 (608687)	Low signal of the dentate due to calcifications in T_2_ or T_2*_—weighted images	[50]
Recessively inherited	CTX(213700)	Hyperintense peridentate and cerebral WM in T_2_-weighted images	[51]
	2-Hydroxic Glutaric Aciduria (236792)	Hyperintense cerebral WM in T_2_-weighted images	[52]
	HLD2(608804)	Hyperintense cerebral, cerebellar, brainstem and spinal cord WM in T_2_-weighted images	[53]
	HLD4(612233)	Lack of normal WM myelination	[54]
	LBSL(611105)	Hyperintense cerebral WM and WM tracts in cerebral and cerebellar peduncles, brainstem and spinal cord in T_2_-weighted images	[55]
	Leukoencephalopthy with ataxia (615651)	Hyperintense internal capsule, cerebral peduncles and middle cerebellar peduncles in T_2_-weighted images with restricted diffusion	[56]
	SANDO(607459)	Hyperintense thalami, middle cerebellar peduncle and cerebellar WM in T_2_-weighted images	[57]
	SCAR4/SCA24(607317)	Hyperintense basal ganglia and cerebral WM in T_2_-weighted images	[58]
X-linked inherited	FXTAS (300623)	Hyperintensity of the middle cerebellar peduncles, splenium corpus callosum and of the cerebral WM in T_2_-weighted images	[59]

Number in parenthesis corresponds to OMIM code. **Abbreviations**: ATXPC = ataxia pancytopenia syndrome; CTX = cerebrotendinous xanthomatosis; FXTAS = fragile-X tremor ataxia syndrome; HLD = hypomyelinating leukodystrophy; LBSL = leukoencephalopathy with brainstem and spinal cord involvement and lactate elevation; SANDO = sensory ataxic neuropathy, dysarthria and ophtalmoparesis; SCA = spino-cerebellar ataxia (dominant); SCAR = spino-cerebellar ataxia recessive; SPG5A = spastic paraplegia 5A; SPAX2 = spastic ataxia 2; WM = white matter.

## Data Availability

No unpublished personal data was utilized for the present review. All MR images and their descriptions are available through links indicated in the paper.
